# Safety and effectiveness of diroximel fumarate in relapsing forms of multiple sclerosis: a systematic review and meta-analysis

**DOI:** 10.1007/s10072-025-08140-8

**Published:** 2025-04-14

**Authors:** Haneen Sabet, Mohamed Ahmed Zanaty, Abdelfattah Arafa, Mohamed El-Moslemani, Shereen Mohamed Olama, Mahmoud G. A. Saleh, Abdallah Abbas, Ahmed Z. Obeidat

**Affiliations:** 1 Faculty of Medicine, South Valley University, Qena, Egypt; 2 Faculty of Medicine, Al-Azhar University, Damietta, Egypt; 3 Department of Internal Medicine, College of Medicine, Northern Border University, Arar, Saudi Arabia; 4 Department of Chemistry, College of Science, Northern Border University, Arar, Saudi Arabia; 5 Department of Neurology, Medical College of Wisconsin, 8701 Watertown Plank Rd, Milwaukee, WI 53226 USA

**Keywords:** Diroximel fumarate, Disease-modifying therapies, Relapsing–remitting multiple sclerosis, Multiple sclerosis

## Abstract

**Objective:**

To evaluate the safety and efficacy of Diroximel Fumarate (DRF) in patients with different relapsing forms of MS (RMS) through systematic review and meta-analysis.

**Methods:**

A systematic review and meta-analysis adhering to PRISMA guidelines was conducted. Scopus, PubMed, and Cochrane CENTRAL databases were searched until December 6, 2024, for clinical trials and observational studies on DRF in RMS. Eligibility criteria included studies evaluating DRF’s safety or efficacy, excluding case reports and non-clinical outcomes. The risk of bias was assessed using the Newcastle–Ottawa Scale and ROBINS-I tools. Statistical analyses were performed using OpenMetaAnalyst, focusing on pooled mean differences and incidence rates with 95% confidence intervals.

**Results:**

Seven studies with 3,075 participants were included. The overall persistence rate was 75.6% (95% CI: 63.5%, 87.7%). The discontinuation rate due to safety concerns was 6.1% (95% CI: 4.1%, 8.1%). Lymphocyte count decreased significantly by -355.02 cells/µL (95% CI: -636.71, -73.32). Mild adverse events (AEs) occurred in 33% (95% CI: 18.6%, 47.4%), moderate in 30% (95% CI: -9.9%, 69.9%), and severe in 5% (95% CI: -3.8%, 13.7%). Gastrointestinal (GI) AEs were observed in 17.4% (95% CI: 6%, 28.8%), flushing in 18.5% (95% CI: 5.7%, 31.3%), and lymphopenia in 24.3% (95% CI: 10.2%, 38.4%). The relapse rate was 7.1% (95% CI: -4.8%, 19%).

**Conclusion:**

DRF demonstrates efficacy in reducing relapse rates and offers an improved safety profile compared to its predecessor, Dimethyl Fumarate (DMF), particularly in GI tolerability. However, lymphopenia requires monitoring. Further research is recommended to evaluate long-term safety and efficacy in diverse populations.

**Supplementary Information:**

The online version contains supplementary material available at 10.1007/s10072-025-08140-8.

## Introduction

Multiple sclerosis (MS) is a chronic, immune-mediated disease characterized by inflammation of the central nervous system (CNS), demyelination, and axonal degeneration. It is estimated to affect more than 2.9 million people worldwide. The major subtype of MS, relapsing–remitting multiple sclerosis (RRMS), constitutes approximately 85% of all MS patients [1, 2]. RRMS is manifested by attacks of neurological dysfunction separated by relative or complete recovery periods, called remissions. Although there is no cure, the advent of disease-modifying therapies (DMTs) has significantly improved the management of MS, reducing relapse rates and new brain and spine lesion formation, delaying progression, and mitigating long-term neurological damage [1, 3].

Diroximel fumarate (DRF), a second-generation oral fumarate, has been an important addition to the MS treatment landscape with some differences from dimethyl fumarate (DMF). Both DRF and DMF share the active metabolite monomethyl fumarate (MMF), which is thought to modulate the inflammatory and immune responses central to MS pathogenesis. DRF is better tolerated in the gastrointestinal (GI) than DMF because of its unique chemical structure, resulting in lower methanol production and reduced GI irritation [2, 3]. This makes DRF a preferable agent, especially for those patients who cannot tolerate DMF because of GI-related adverse events (AEs) [2, 4]. Safety, tolerability, and efficacy have been studied in detail for DRF in clinical and real-world settings. The phase 3 EVOLVE-MS-1 trial showed that DRF exhibits sustained safety, with very low discontinuation rates for AEs, and it induced significant reductions in relapse rates over two years [5]. These observations are further supported by the real-world data that indicates high persistence and adherence rates to this DRF, even among patients transitioning from other DMTs [3, 4]. Besides, DRF has demonstrated some potential in maintaining lymphocyte counts within safety limits, which is crucial in minimizing opportunistic infections, including progressive multifocal leukoencephalopathy [2, 3].

Despite these advances, challenges persist in optimizing treatment strategies for relapsing forms of MS (RMS). Patient tolerability, perceived efficacy of treatment, and regimen complexity are factors that continuously affect adherence, persistence with, and overall effectiveness of DMTs [4, 6]. The current systematic review and meta-analysis will, therefore, collate evidence on the clinical outcomes of DRF in RMS, providing a comprehensive assessment of its safety and efficacy profiles. By integrating insights from controlled trials and observational studies, we seek to delineate the role of DRF in enhancing patient care and improving long-term neurological outcomes.

## Methods

We carried out this study according to the Preferred Reporting Items of Systematic Reviews and Meta-Analyses (PRISMA) and the Cochrane Handbook for Systematic Reviews of Interventions [7, 8].

### Search and eligibility criteria

A comprehensive search strategy was implemented using Scopus, PubMed, and Cochrane CENTRAL databases, covering all records from inception up to December 6, 2024. The search terms included: (("Diroximel fumarate" OR DRF OR Vumerity) *AND* ("Multiple Sclerosis" OR MS OR "Relapsing–Remitting Multiple Sclerosis" OR RRMS)).

We included observational studies and clinical trials, both single-arm and double-arm designs that evaluated the safety or efficacy of DRF in patients with RMS. Exclusion criteria encompassed editorials, abstracts, case reports, case series, studies focusing on unrelated outcomes such as pharmacokinetics, and studies involving interventions other than DRF.

### Screening and data extraction

Three authors independently conducted the screening using the Rayyan software [9] in two phases. In the first phase, titles and abstracts were screened. In the second phase, full texts of potentially relevant studies were reviewed. Additionally, references of included studies were screened for further relevant studies. Any disagreements during the screening process were resolved through discussion and referral to the first author.

Data extraction was carried out independently by three authors using Microsoft Excel [10], with any discrepancies resolved by the first author. The data extraction included:

(i) **Summary of included studies**: Variables such as study ID, country, duration, study design, population, intervention protocol, outcomes measured, and study summary.
(ii) **Baseline characteristics**: Variables such as sample size, age, sex, race, disease duration, DRF treatment duration, and prior DMT.
(iii) **Outcomes**: Persistence rate, *safety profile* (discontinuation rates due to lack of safety, lymphocyte count, mild AEs, moderate AEs, severe AEs, GI symptoms, flushing, and lymphopenia), and *efficacy profile* (relapse rates).

### Risk of bias assessment

We conducted the evaluation of bias risk using the Newcastle–Ottawa Scale (NOS) tool [11]. This tool reviews each observational study based on nine criteria that are organized into three groups: participant selection, group comparability, and the determination of either the exposure or outcome of interest. Each criterion is rated as either 'yes' or 'no'. Studies that achieve a score between 7 and 9 are classified as high quality, while those scoring between 5 and 6 are regarded as fair quality. Scores that fall between 1 and 4 are identified as poor quality.

For Non-Randomized Studies of Interventions (NRSI), we employed the Risk of Bias In Non-randomized Studies (ROBINS-1) tool to evaluate bias [12]. This tool examines seven domains of bias: confounding bias, selection bias, bias due to intervention classification, deviations from intended interventions, loss of data, outcome measurement bias, and bias in reported results selection. The overall quality of the study is assessed by analyzing the risk of bias across each domain, leading to an overall judgment of the risk of bias regarding the outcome being evaluated. This judgment is categorized as low risk, moderate risk, serious risk, or critical risk of bias.

### Statistical analysis

We used the OpenMetaAnalyst software to analyze our data [13]. The pooled mean difference (MD) and 95% confidence interval (CI) were used for continuous data and the pooled incidence rate and 95% CI for categorical data, respectively. To calculate the overall effect size for all outcomes, we applied the DerSimonian and Laird random effects model [14]. This method gives more weight to smaller research and describes the included studies as a random sample drawn from a bigger population. We choose this model because it can handle more variability in the pooled estimate, which makes it appropriate for data that can show differences or inconsistencies. As a result, our meta-analysis's results yield conservative estimates that take these possible discrepancies into consideration.

The chi-squared test and the I-squared (I^2^) statistic were used to assess statistical heterogeneity among the studies. Significant heterogeneity was present when the I^2^ value was greater than 50% or the P-value was less than 0.1 [15]. Given the small number of studies included in each analysis (fewer than 10), we could not reliably conduct Egger’s regression test for publication bias or perform meta-regression in accordance with Cochrane guidelines [16].

## Results

### Search and screening

**Fig. 1:**
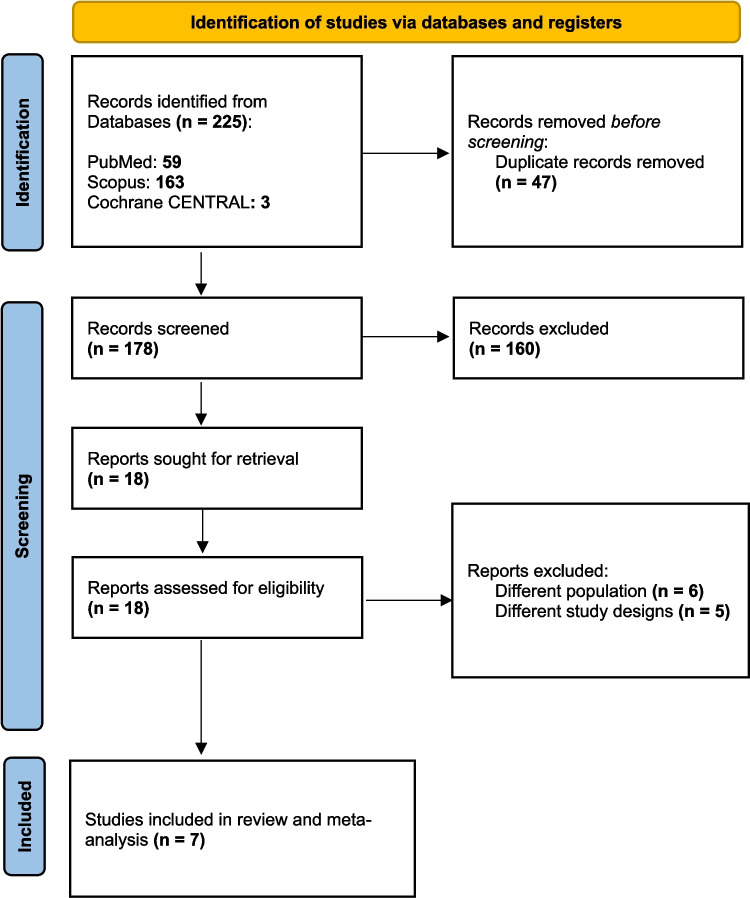
PRISMA flow diagram of search and screening process

The initial database search identified 225 articles. After removing duplicates, the count was reduced to 178. Subsequent title and abstract screening further narrowed the selection to 18 articles. Following a full-text review, seven studies [1–6, 17] were deemed eligible for inclusion (see Fig. 1).

### Summary and baseline characteristics

The meta-analysis included seven studies with a total of 3,075 participants who received DRF for RMS. Among the participants, 759 (24.67%) were male, and 2,316 were female. The overall pooled mean age across the studies was approximately 47.4 years. Tables 1 and 2 summarize detailed data on sample size, demographic characteristics, and treatment durations for each study. These tables also provide additional insights into variables such as race, disease duration, prior DMTs, and the average duration of DRF treatment, which differed between studies.

**Table 1:** Summary of the included studies

Study ID	Country	Duration	Study design	Population	Intervention protocol	Outcomes measured	Summary of the study
Aguire 2024	Spain	November 2022 to October 2023	Multicenter, prospective observational study	MS patients who started treatment with DRF in participating clinics	Patients were treated with DRF for a mean duration of 6.3 months (SD: 7.3)	Safety (AEs, lymphocyte counts, lymphopenia rates), Efficacy (relapse rates, EDSS progression, radiological activity)	Most patients (87.3%) improved in side effects (GI, flushing) compared to DMF. Relapse rates were low (1.6%), with EDSS stability or improvement in > 90%. Most continued DRF, confirming its tolerability and safety. Minimal safety concerns, with 6 cases of mild lymphopenia requiring discontinuation
Araujo 2022	USA	January 2020 to June 2021	Retrospective real-world database study	MS patients newly initiated teriflunomide, DMF, DRF, or fingolimod between January 1, 2020, and June 30, 2020	Oral DMTs, including DRF, twice daily, between January 1, 2020, and June 30, 2020	Persistence, Adherence, Switching to higher-cost therapies (ocrelizumab, natalizumab, cladribine)	Among 6934 patients, those on Teriflunomide and Fingolimod showed higher persistence (60% and 66%) and adherence (55% and 59% at 12 months) compared to DMF and DRF (persistence: 44% and 49%; adherence: 40% and 44%). DRF patients were among those with the shortest time to switch to higher-cost therapies (e.g., 247 days to natalizumab)
Dempsey 2024	USA	August 2021 to October 2023	Retrospective cohort analysis from medical records	Patients with RRMS clinically stable on DMF, who switched to DRF	Switch from DMF at 240 mg twice daily to DRF at 462 mg twice daily	Lymphopenia (grades, absolute lymphocyte count), Absolute T-cell subsets (CD3 + , CD4 + , CD8 +), AEs	Of the 22 patients switching from DMF to DRF, 63.64% experienced an immediate decrease in ALC. Grade 3–4 lymphopenia occurred in 22.73% of DRF patients, compared to none on DMF. Adverse events led to discontinuation of DRF in 40.91% of the cohort
Gudesblatt 2024	USA	1 Year	Observational study	Clinically stable RRMS adults on Ocrelizumab (EDSS 0–6.5, ≥ 1 year)	DRF was administered for 1 year after a 4.2 to 18.4-month washout period from last dose of Ocrelizumab	Relapse rate, MRI, peripheral immune parameters, CAB, OCT, PROs	A year after switching from Ocrelizumab to DRF, no relapses were reported, no significant changes in immune parameters except for an increase in CD19 + cell percentage, and no significant changes in CAB, OCT, and PROs
Gudesblatt 2022	USA	October 2020 to December 2020	Observational study	Adult MS patients taking DRF for at least 3 weeks in the past 6 months	DRF, treatment duration ranged from 6 weeks to 10 months	Patient Perceptions: Overall wellness, quality of life, ease of administration, manageable side effects, and patient optimism. Safety: AEs	Patients reported overall wellness, ease of administration, and minimal side effects as a positive result of DRF treatment. Though Intense treatment regimen and food requirements were negative sides of the drug
Larger 2023	USA	December 2019 to January 2021	Retrospective analysis	Patients with MS who received DRE from AcariaHealth specialty pharmacy provider	The patients started taking DRF and they had the potential to be treated with DRF for 5 months	Persistence, Discontinuation rate due to GI AEs, and Adherence	Of 1143 patients in overall group and 433 in "DMF to DRF" group, most (90.1%) were able to persist on DRF in "DMF to DRF" group and (82.3%) in overall group and most of them were able to tolerate DRF (90.8%) in overall group and (90.7%) in "DMF to DRF" group
Singer 2023	USA, Canada, Europe	December 2015 to November 2021	Open-label single arm clinical trial (phase 3)	RRMS patients, neurologically stable in the 30-day prior	DRF 462 mg twice daily for 96 weeks and it was titrated for newly enrolled patients	Safety, Tolerability, Lymphocytic Count, Relapsing Rate, Radiological endpoint, T2, and PROs	Most patients persisted on DRF (75.7% overall, 82.5% newly diagnosed). Relapse rates were low (17.6% overall, 15.6% newly diagnosed). Patients tolerated DRF well. Lymphocyte counts decreased in De novo enrollment, Prior DMF, and Prior DRF groups (−25.7, −27.6, and −30.3, respectively)

*AEs* Adverse Events; *ALC* Absolute Lymphocyte Count; *CAB* Cognitive Assessment Battery; *MRI* Magnetic Resonance Imaging; *DMF* Dimethyl Fumarate; *DMTs* Disease-Modifying Therapies; *DRF* Diroximel Fumarate; *EDSS* Expanded Disability Status Scale; *GI* Gastrointestinal; *MS* Multiple Sclerosis; *OCT* Optical Coherence Tomography; *PROs* Patient-Reported Outcomes; *RRMS* Relapsing–Remitting Multiple Sclerosis

**Table 2:** Baseline characteristics

Study ID	Sample size (n)	Age, years, mean (SD)	Sex, Males/ Females	Race	Duration of the disease (time till diagnosis), years, Mean (SD)	Diroximel fumarate duration, months, Mean (SD)	Prior disease-modifying therapy (DMT), Name (%)
Aguire 2024	195	42.2 (9.4)	40/155	NA	10.2 (8.7)	6.3 (7.3)	Dimethyl Fumarate (DMF): (61.9%), DMT other than DMF: (7.2%)
Araujo 2022	616	49 (11.8)	121/495	NA	NA	Up to 12 months	Treatment-naïve: (38%), DMT: (63%)
Dempsey 2024	22	48.75 (10.211)	5/17	White: 18 (81.8%), Non-White: 4 (18.18%)	13 (8.6)	15 (4.6)	Glatiramer Acetate (4.55%), Teriflunomide (4.55%), Interferon β 1a (22.73%), Natalizumab (4.55%)
Gudesblatt 2024	25	52 (9)	9/16	NA	10.2 (8.4)	12	Ocrelizumab (100%)
Gudesblatt 2022	17	49.3 (12)	6/11	White: 16 (94.1%), Hispanic/Latino: 1 (5.9%)	Range: 1 to 29	Range: 1.5 to 10	Dimethyl fumarate: (58.8%), Glatiramer acetate: (41.2%), Interferon β−1a IM injection: (29.4%), Ocrelizumab: (29.4%), Natalizumab: (29.4%), Interferon β−1b: (17.6%), Interferon β−1a SC injection: (17.6%), Teriflunomide: (5.9%), Fingolimod: (5.9%), Rituximab: (5.9%)
Larger 2023	1143	51 (9.8)	283/860	NA	NA	8.575 (3.047)	Interferon (2.4%), Glatiramer acetate (0.4%), Teriflunomide (0.3%), Siponimod (0.1%), Natalizumab (0.1%), DMF (37.9%)
Singer 2023	1057	42.5 (10.8)	295/762	White: 972 (92%), Black or African American: 72 (6.8%), Others: 13 (1.2%)	7.6 (7.3)	16 (3.696)	Interferon (37.7%), Glatiramer acetate (25.3%)

*DMT* Disease-Modifying Therapy; *DMF* Dimethyl Fumarate; *IM* Intramuscular; *SC* Subcutaneous

### Risk of bias assessment

Six studies were evaluated using the NOS tool. Five of the studies [1, 2, 4, 6, 17] demonstrated overall good quality, while one study was rated as having overall fair quality (see Supplementary Table 1). Additionally, one study [5] was assessed using the ROBINS-I tool and received an overall moderate quality rating (see Supplementary Table 2).

### Meta-analysis

#### Persistence rate

**Fig. 2:**
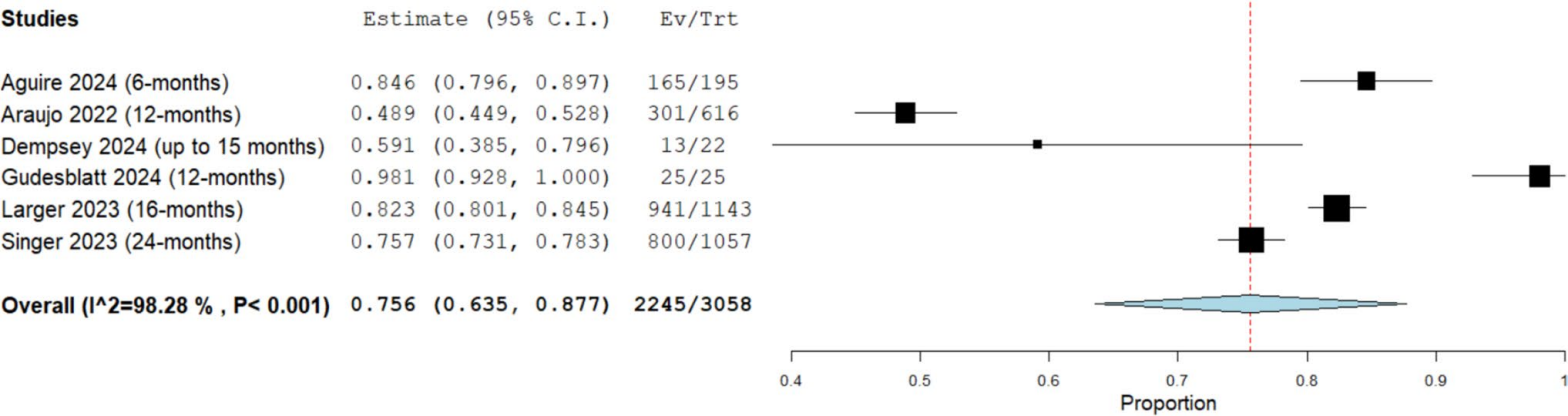
Overall persistence rate in patients taking diroximel fumarate (DRF)

The overall persistence rate in a cohort of 3,058 patients, derived from six studies, with a mean follow up duration of 14.17 months (range: 6 to 24 months), was determined to be 75.6% (95% CI: [63.5%, 87.7%], see Fig. 2). Notably, there was significant heterogeneity among the studies (I^2^ = 98%, *P* < 0.001). Heterogeneity was not resolved after the leave-one-out analysis. This finding suggests that, on average, seven to eight out of every ten patients treated with DRF demonstrated treatment persistence.

#### Safety profile

##### Discontinuation rate due to lack of safety

**Fig. 3:**
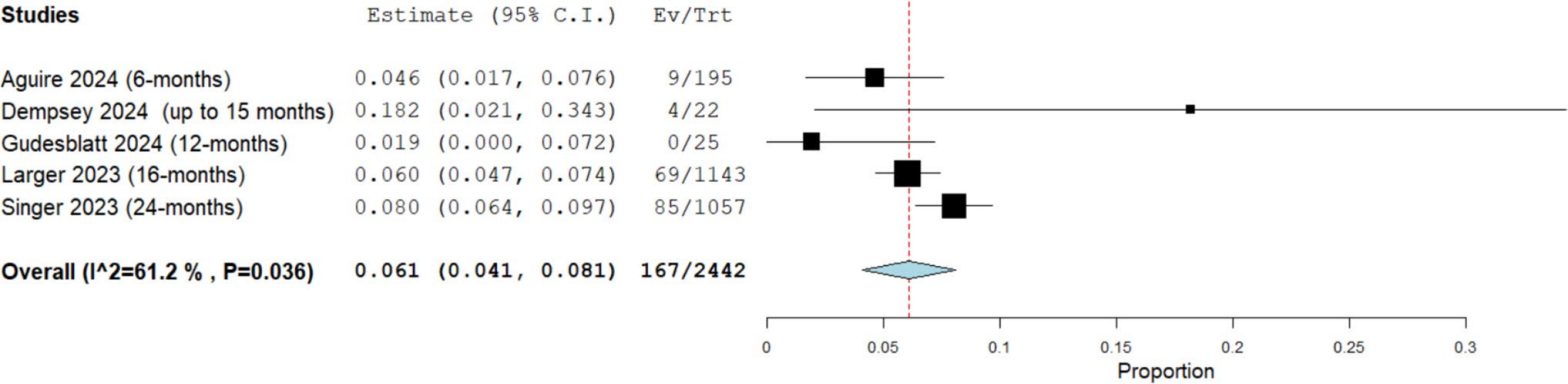
Overall discontinuation rate due to lack of safety in patients taking diroximel fumarate (DRF)

The overall discontinuation rate due to safety concerns in a cohort of 2,442 patients, derived from five studies, with a mean follow-up duration of 14.6 months (range: 6 to 24 months), was 6.1% (95% CI: [4.1%, 8.1%]; see Fig. 3). Moderate heterogeneity was observed among the studies (I^2^ = 61%, P = 0.036). This finding indicates that, on average, approximately one in every ten patients treated with DRF discontinued the therapy due to safety-related issues. To address this heterogeneity, we conducted a leave-one-out analysis, which revealed that Singer 2023 [5] was the source of heterogeneity (Supplementary Fig. 1**)**.

##### Reduction in lymphocyte count

**Fig. 4:**
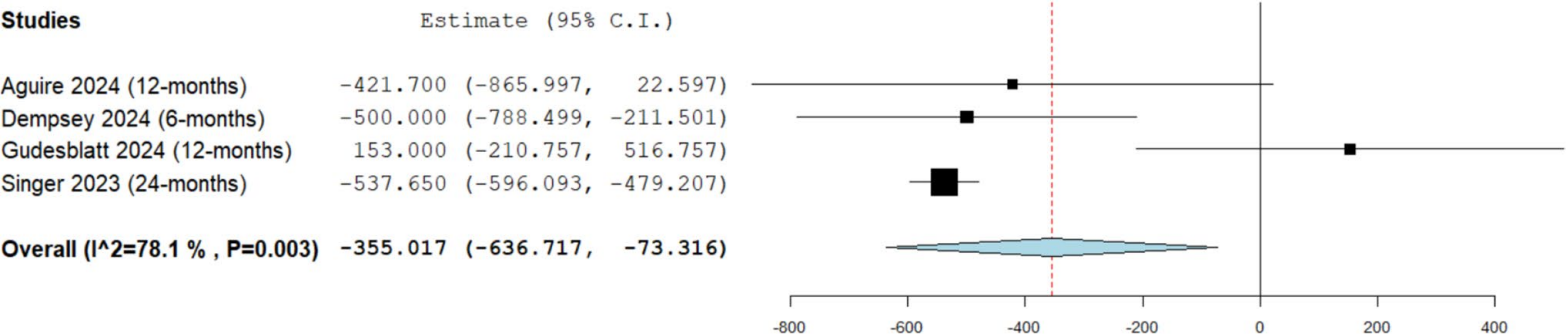
Mean decrease in lymphocyte count after diroximel fumarate (DRF) treatment

A statistically significant reduction in lymphocyte count was observed following treatment with DRF compared to baseline (MD: −355.02, 95% CI: [−636.72, −73.32]; see Fig. 4), with a mean follow-up duration of 13.5 months (range: 6 to 24 months). The data showed substantial heterogeneity (I^2^ = 78%, P = 0.003). This finding indicates that, on average, patients treated with DRF experienced a significant reduction in lymphocyte counts by 355.02 cells/μL, highlighting the importance of periodic monitoring for potential safety-related concerns. To address this heterogeneity, we conducted a leave-one-out analysis, which revealed that Gudesblatt 2024 [2] was the source of heterogeneity (Supplementary Fig. 2).

##### Mild AEs

**Fig. 5:**
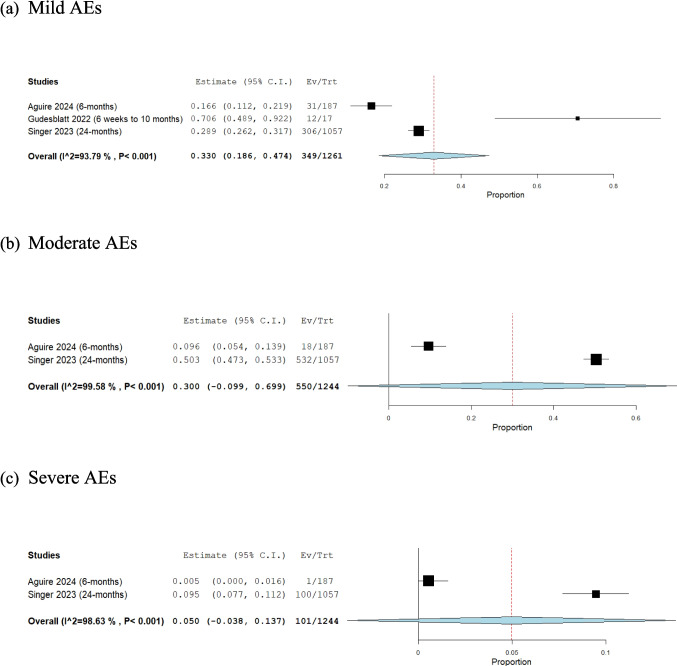
Mild, moderate, and severe adverse events (AEs) in patients taking diroximel fumarate (DRF), (**a**) Mild AEs (**b**) Moderate AEs, (**c**) Severe AEs

The overall mild AEs rate in a cohort of 1,261 patients, derived from three studies, with a mean follow-up duration of 11.9 months (range: 1.5 to 24 months), was 33% (95% CI: [18.6%, 47.4%]; see Fig. 5A). Significant heterogeneity was observed among the studies (I^2^ = 93%, P < 0.001). However, a leave-one-out analysis was not applicable due to the inclusion of only two studies. This finding indicates that, on average, approximately three to four in every ten patients treated with DRF experienced mild AEs.

##### Moderate AEs

The overall moderate AEs rate in a cohort of 1,244 patients, derived from two studies,with a mean follow-up duration of 15 months (range: 6 to 24 months), was 30% (95% CI: [9.9%, 69.9%]; see Fig. 5B). Significant heterogeneity was observed among the studies (I^2^ = 99.6%, P < 0.001). However, a leave-one-out analysis was not applicable due to the inclusion of only two studies. This finding indicates that, on average, approximately three in every ten patients treated with DRF experienced moderate AEs.

##### Severe AEs

The overall severe AEs rate in a cohort of 1,244 patients, derived from two studies, with a mean follow-up duration of 15 months (range: 6 to 24 months), was 5% (95% CI: [3.8%, 13.7%]; see Fig. 5C). Significant heterogeneity was observed among the studies (I^2^ = 98.6%, *P* < 0.001). However, a leave-one-out analysis was not applicable due to the inclusion of only two studies. This finding indicates that, on average, approximately one in every ten patients treated with DRF had severe AEs.

##### GI AEs

**Fig. 6:**
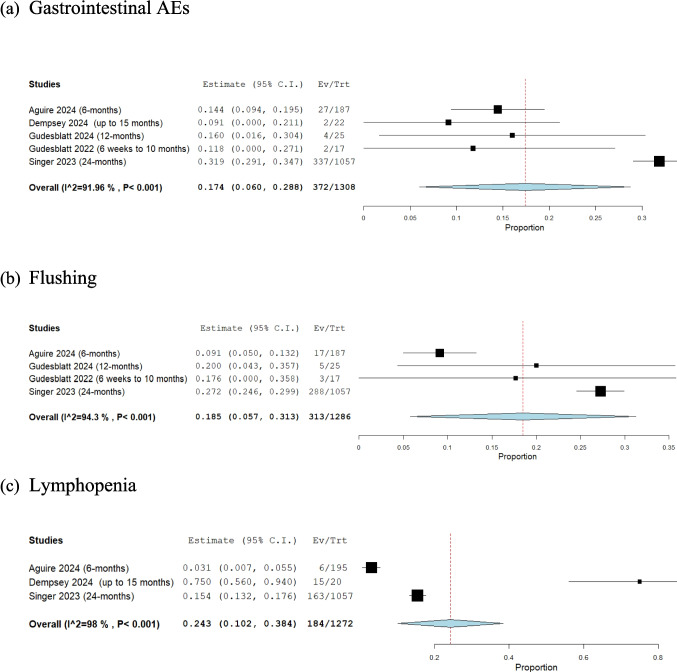
Gastrointestinal (GI), flushing, and lymphopenia adverse events (AEs) in patients taking diroximel fumarate (DRF), (**a**) Gastrointestinal (GI) AEs, (**b**) Flushing, (**c**) Lymphopenia

The overall rate of GI AEs in a cohort of 1,308 patients, derived from five studies, with a mean follow-up duration of 12.55 months (range: 1.5 to 24 months), was 17.4% (95% CI: [6%, 28.8%]; see Fig. 6A). Significant heterogeneity was observed among the studies (I^2^ = 91.9%, *P* < 0.001). This finding indicates that, on average, approximately two in every ten patients treated with DRF experienced GI AEs. To address this heterogeneity, we conducted a leave-one-out analysis, which showed that Singer 2023 [5] was the source of heterogeneity. (Supplementary Fig. 3).

##### Flushing

The overall rate of flushing in a cohort of 1,286 patients, derived from four studies, with a mean follow-up duration of 12 months (range: 1.5 to 24 months) was 18.5% (95% CI: [5.7%, 31.3%]; see Fig. 6B). Significant heterogeneity was observed among the studies (I^2^ = 94.3%, *P* < 0.001). This finding indicates that, on average, approximately two in every ten patients treated with DRF experienced flushing. To address this heterogeneity, we conducted a leave-one-out analysis, which showed that Aguire 2024 [1, 2] was the source of heterogeneity. (Supplementary Fig. 4).

##### Lymphopenia

The overall rate of lymphopenia in a cohort of 1,272 patients, derived from three studies, with a mean follow-up duration of 15 months (range: 6 to 24 months), was 24.3% (95% CI: [10.2%, 38.4%]; see Fig. 6C). Significant heterogeneity was observed among the studies (I^2^ = 98.6%, *P* < 0.001). Heterogeneity was not resolved after the leave-one-out analysis. Among the 184 patients who developed lymphopenia, 12 had grade 1 or 2, nine had grade 3 or 4, and the grade was not specified for the remaining cases. This finding indicates that, on average, approximately two to three in every ten patients treated with DRF experienced lymphopenia.

#### Efficacy profile

##### Relapse rate

**Fig. 7:**
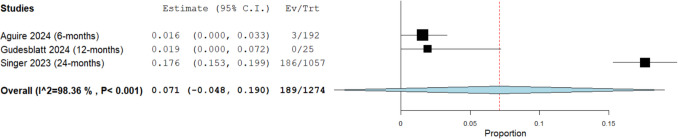
Overall relapse rate in patients taking diroximel fumarate (DRF)

The overall relapse rate in a cohort of 1,274 patients, derived from three studies, with a mean follow-up duration of 14 months (range: 6 to 24 months) was 7.1% (95% CI: [4.8%, 19%]; see Fig. 7). Significant heterogeneity was observed among the studies (I^2^ = 98.4%, *P* < 0.001). This finding indicates that, on average, one in ten patients treated with DRF experienced a relapse during the study periods. To address this heterogeneity, we conducted a leave-one-out analysis, which showed that Singer 2023 [5] was the source of heterogeneity. (Supplementary Fig. 5).

## Discussion

To our knowledge, this is the first systematic review and meta-analysis to thoroughly examine the efficacy and safety of DRF in RMS. The results suggest that DRF can be an effective and well-tolerated therapy for managing RMS. On average, seven to eight out of every ten patients treated with DRF remained persistent, with a mean follow-up duration of 14.17 months (range: 6 to 24 months), highlighting strong long-term adherence in real-world clinical settings.

From a safety perspective, mild AEs were reported in 33% of patients (mean follow-up: 11.9 months, range: 1.5 to 24 months), moderate AEs in 30% (mean follow-up: 15 months, range: 6 to 24 months), and severe AEs in 5% (mean follow-up: 15 months, range: 6 to 24 months), with severe events being relatively uncommon. Notably, GI-related AEs affected 17.4% (mean follow-up: 12.55 months, range: 1.5 to 24 months), while flushing was observed in 18.5% (mean follow-up: 12 months, range: 1.5 to 24 months); however, these issues were generally manageable. Importantly, lymphopenia occurred in 24.3% (mean follow-up: 15 months, range: 6 to 24 months), emphasizing the need for regular monitoring to mitigate potential risks. Additionally, DRF treatment led to a significant mean reduction in lymphocyte count by −355.02 cells/μL (mean follow-up: 13.5 months, range: 6 to 24 months).

In terms of efficacy, DRF demonstrated promise in reducing disease activity, with a relapse rate of 7.1% (mean follow-up: 14 months, range: 6 to 24 months), indicating that only one in ten patients experienced a relapse during the study period. These findings highlight DRF’s potential to balance effectiveness and safety, making it a compelling option for managing RMS.

Data, in general, were consistent between available evidence from pivotal clinical trials and real-world studies, but at times, we observed discrepancies that require further investigation. High persistence rates observed in this meta-analysis align with findings from Lager et al. [3], where more than 90% of patients transitioning from DMF to DRF remained adherent. However, frequent dosing and dietary restrictions may challenge adherence, as revealed through patient interviews by Gudesblatt et al. [6]. These findings highlight the importance of tailoring treatment regimens to individual preferences and lifestyles.

In the EVOLVE-MS-1 trial [5], an adjusted annualized relapse rate (ARR) of 0.13 was reported, consistent with the low relapse rates observed in this meta-analysis. Additionally, the trial demonstrated reductions in gadolinium-enhancing lesions and stable or improved Expanded Disability Status Scale (EDSS) scores over two years, reinforcing the disease-modifying potential of DRF as shown in phase 3 data by Araujo et al. [4] Real-world evidence from Aguirre et al. and Lager et al. [1, 3] also supports low relapse rates in DRF-treated patients, with stable magnetic resonance imaging (MRI) outcomes and reduced disease activity. However, studies like Araujo et al. [4] suggest lower persistence with DRF than teriflunomide and fingolimod, highlighting the need for a nuanced understanding of patient preferences and barriers to adherence.

GI AEs remain a key concern with fumarate therapies. DRF’s improved tolerability compared to DMF is attributed to its unique chemical structure, which limits methanol production and reduces GI irritation. These findings are supported by data from the EVOLVE-MS-2 study [5], which showed fewer and less severe GI AEs with DRF than DMF. However, lymphopenia remains a risk for both DRF and DMF. Dempsey et al. [17] noted a potential worsening of lymphopenia in patients transitioning from DMF to DRF, raising concerns about the long-term safety of DRF and the need for close monitoring, especially during treatment transitions.

Recent meta-analyses of DMF in MS (2020–2024) reinforce its efficacy in reducing relapse activity, showing significant drops in ARR (on the order of –0.2 per year) and roughly a halving of relapse risk versus placebo [18]. These reviews also underscore DMF’s well-known safety/tolerability profile: flushing (in ~ 30–40% of patients) and GI AEs (e.g. nausea and diarrhea in ~ 10–20%) [18] are common and can lead to treatment cessation in a subset of patients (about 9% discontinuing due to GI intolerance) [19]. Indeed, a 2020 systematic review of 12,380 DMF-treated patients found higher overall AE rates with DMF than placebo (Risk Ratio [RR] ≈1.37) but no increase in serious AEs; notably, severe lymphopenia occurred in ~ 4% of patients, necessitating regular monitoring [19]. Despite these issues, DMF’s persistence on therapy remains moderate—many patients continue treatment beyond 1–2 years, though tolerability challenges (GI or laboratory AEs) account for a proportion of dropouts. In contrast, the present meta-analysis of DRF indicates comparable efficacy in relapse reduction alongside improved tolerability. DRF-treated cohorts achieved similarly low relapse rates comparable to DMF outcomes [18], while experiencing fewer GI-related AEs (~ 17% incidence) and less frequent flushing (~ 18%). This translated into a higher persistence rate on DRF (~ 75% of patients remained on therapy) with fewer discontinuations for side effects (~ 6% due to AEs)—aligning with reports of DRF’s favorable GI profile and lower dropout rates in clinical trials. Both fumarate formulations share class-related risks (e.g., lymphocyte count reductions) requiring surveillance [19], but no new safety concerns have emerged with DRF to date. It is important to note differences in follow-up: DMF’s efficacy and safety outcomes were typically measured at 24-month endpoints in pivotal trials [18], whereas DRF’s current evidence base stems from shorter observation periods (including a 24-week head-to-head GI tolerability study and open-label extensions up to ~ 96 weeks). This discrepancy in timeframe tempers direct comparisons of long-term effects; however, early data suggest that DRF provides similar disease control to DMF with a more favorable safety and tolerability profile in the short-to-medium term, particularly with respect to GI AEs [19].

The efficacy of DRF is primarily linked to its active metabolite, MMF, which is thought to provide anti-inflammatory effects and minimize CNS infiltration by immune cells [20]. DRF's ability to sustain high persistence rates, even among patients switching from other therapies, reflects a safety profile that positively impacts long-term adherence. Nonetheless, the risks associated with lymphopenia and other AEs emphasize the need for careful patient selection and ongoing monitoring. As noted by Araujo et al., variations in adherence rates between DRF and alternative treatments like teriflunomide and fingolimod [4] may arise from differences in dosing schedules and perceived efficacy. Future research should examine these aspects to develop strategies for improving adherence among diverse patient populations.

### Strengths and limitations

This meta-analysis integrates diverse study designs, populations, and outcomes, comprehensively assessing DRF’s clinical utility. However, several limitations must be acknowledged. Firstly, variations in methodologies, follow-up durations, and patient demographics across studies introduce potential biases. For example, real-world adherence rates for DRF varied significantly depending on the study setting and population [1, 3]. Secondly, most included studies focused on Western populations, limiting the generalizability of findings to diverse demographic and genetic backgrounds [5]. Also, different outcomes like the treatment discontinuation rates and relapse rate were reported over different follow-up periods, which may affect the comparability of outcomes. While this analysis confirms DRF’s short- to medium-term safety and efficacy, long-term outcomes, particularly regarding lymphopenia, remain underexplored [17].

### Implications for clinical practice

These findings highlight the efficacy and tolerability of DRF as a treatment option for RMS. DRF’s improved GI tolerability makes it particularly beneficial for patients who experience AEs with DMF. Furthermore, its favorable safety profile supports its use in de-escalation strategies during periods of increased immune suppression risk, such as the COVID-19 pandemic. The significance of personalized care cannot be overstated. Treatment decisions should be guided by factors such as immune function, comorbid conditions, and patient preferences. Regular monitoring of lymphocyte counts and patient-reported outcomes is essential to maximize therapeutic benefits while mitigating risks.

### Future research directions

To address current knowledge gaps, future research should prioritize the following areas:

- **Long-Term Safety:** Assessing the progression of lymphopenia and its clinical implications through extended follow-up studies.
- **Direct Comparisons with High-Efficacy Therapies:** Conduct head-to-head trials or propensity score matching studies to compare DRF with high-efficacy alternatives like anti-CD20 monoclonals, natalizumab, or cladribine to better understand its relative safety and effectiveness.
- **Patient-Centric Metrics:** Including broader quality of life measures and real-world adherence data to gain deeper insights into patient experiences and preferences.
- **Diverse Populations:** Expanding studies to include underrepresented populations to ensure equitable treatment outcomes across diverse demographic and genetic groups.
- **De-Escalation** **Studies**: To study de-escalation from anti-CD20 to DRF or other fumarates for patients with increased risk of infections and other complications due to prolonged immunosuppression and B cell depletion.

## Conclusion

This systematic review and meta-analysis suggest that DRF may be an effective and well-tolerated DMT for RMS. The findings indicate high persistence rates associated with DRF, which may reflect its improved tolerability profile, particularly in relation to GI AEs. Additionally, the low relapse rates observed across the included studies provide preliminary evidence of its potential efficacy in reducing disease activity.

Nonetheless, safety concerns, including lymphopenia, emphasize the importance of ongoing patient monitoring and careful selection of candidates for DRF therapy. Although DRF appears to offer improved tolerability compared to DMF, variability in adherence and outcomes highlights the need for more tailored treatment approaches.

Further research is warranted to clarify DRF's long-term safety profile, particularly regarding immune-related risks, and assess its comparative efficacy against other high-efficacy therapies. Broader studies incorporating diverse populations and patient-centric metrics could provide deeper insights into DRF’s potential role in managing RMS and improving patient outcomes.

## Supplementary Information

Below is the link to the electronic supplementary material.

Supplementary file1 (DOCX 90 KB)

## Data Availability

This systematic review and meta-analysis relied on publicly available data from previously published studies. The original research contributions utilized in this study can be accessed within the main article and supplementary materials.

## Declarations

### Previous publication

We declare that this research paper has not been published elsewhere in any other academic journal or platform.

### Generative AI in scientific writing

We declare that we have not used AI in writing any part of this manuscript.
