# Supplementary material for: Safety and effectiveness of diroximel fumarate in relapsing forms of multiple sclerosis: a systematic review and meta-analysis
Source: Neurol Sci. 2025 Apr 14;46(8):3477–90. doi: 10.1007/s10072-025-08140-8 (PMC12267325; doi:10.1007/s10072-025-08140-8)

**Supplementary Table 1.** Risk of bias assessment for observational studies using the NOS tool

| **Study ID** | **Selection** | | | | **Comparability** | **Outcome** | | | **Total (maximum of nine stars)** |
| --- | --- | --- | --- | --- | --- | --- | --- | --- | --- |
|  | Representativeness of the exposed cohort | Selection of the non-exposed cohort | Ascertainment of exposure | The outcome of interest was not present at the start of the study |  | Assessment of outcome | Was follow-up long enough for outcomes to occur | Adequacy of follow-up of cohorts |  |
| **Aguirre 2024** | ⭐ | NA | ⭐ | ⭐ | NA | ⭐ | ⭐ | ⭐ | 6 out of 6 (Good) |
| **Dempsy 2024** | ⭐ | ⭐ | ⭐ | ⭐ | ⭐ | ⭐ | ⭐ | ⭐ | 8 out of 9 (Good) |
| **Gudesblatt 2024** | 0 | NA | ⭐ | ⭐ | NA | ⭐ | ⭐ | ⭐ | 5 out of 6 (Good) |
| **Lager 2023** | ⭐ | NA | ⭐ | 0 | NA | ⭐ | ⭐ | 0 | 4 out of 6 (Fair) |
| **Araujo 2022** | ⭐ | 0 | ⭐ | ⭐ | ⭐ | ⭐ | ⭐ | ⭐ | 7 out of 9 (Good) |
| **Gudesblatt 2022** | ⭐ | NA | ⭐ | ⭐ | NA | ⭐ | ⭐ | ⭐ | 6 out of 6 (Good) |

**Supplementary Table 2.** Risk of bias assessment for non-randomized clinical trials using the ROBINS-1 tool

| **Study** | **D1** | **D2** | **D3** | **D4** | **D5** | **D6** | **D7** | **Overall** |
| --- | --- | --- | --- | --- | --- | --- | --- | --- |
| **Singer 2023** | Moderate | Low | Low | Moderate | Moderate | Low | Low | Moderate |

**D1** Bias due to confounding; **D2** Bias in the selection of participants into the study; **D3** Bias in the classification of interventions; **D4** Bias due to deviations from intended interventions, **D5** Bias due to missing data; **D6** Bias in the measurement of outcomes; **D7** Bias in the measurement of outcomes

**Supplementary Figure 1.** Leave one out analysis of discontinuation rate due to lack of safety


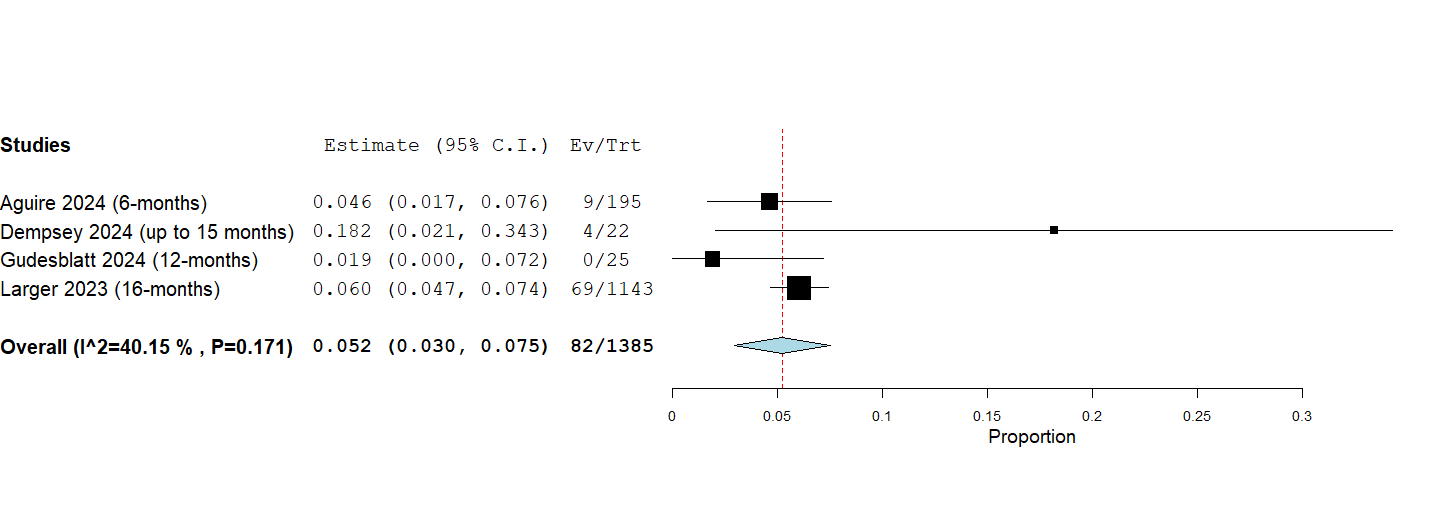


**Supplementary Figure 2.** Leave one out analysis of mean decrease in lymphocyte count after diroximel fumarate (DRF) treatment


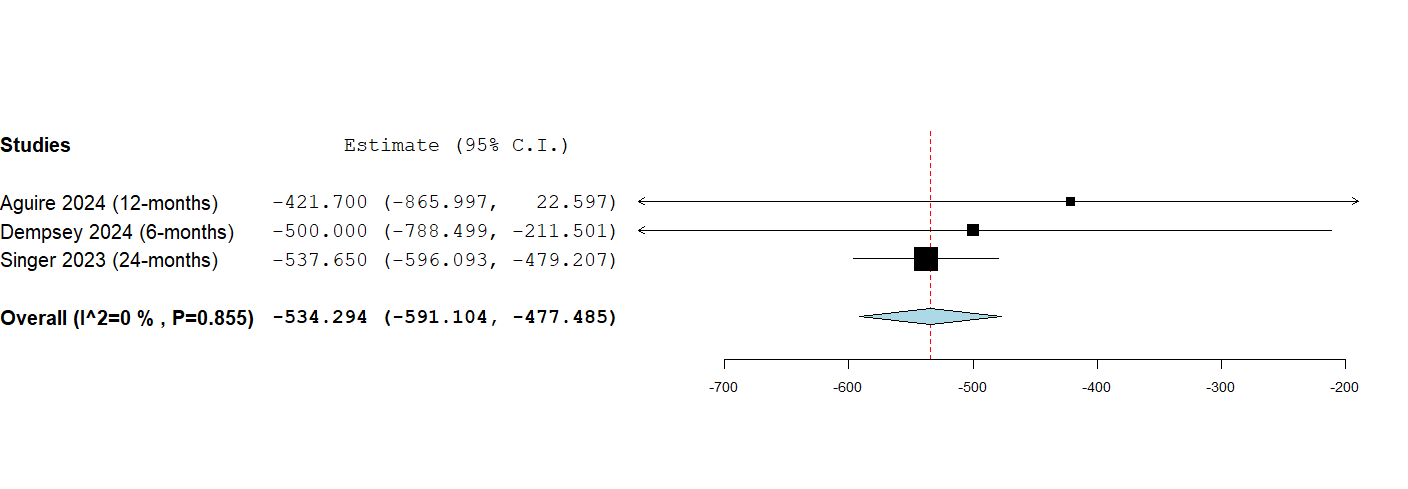


**Supplementary Figure 3.** Leave one out analysis of gastrointestinal adverse events


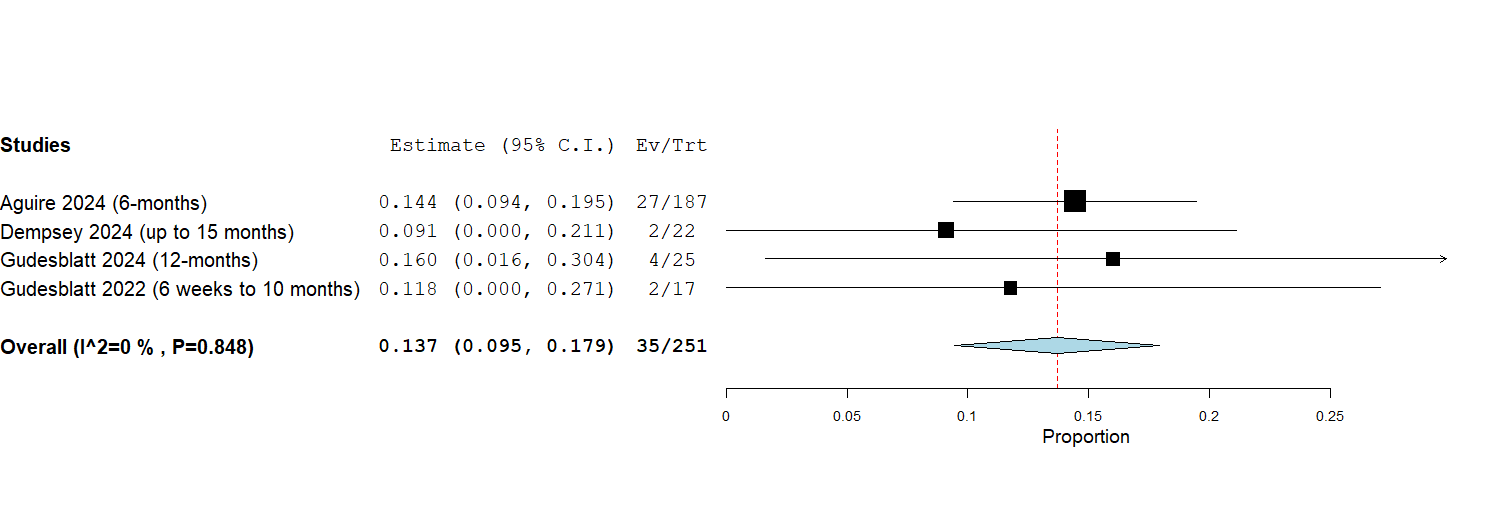


**Supplementary Figure 4.** Leave one out analysis of flushing adverse event


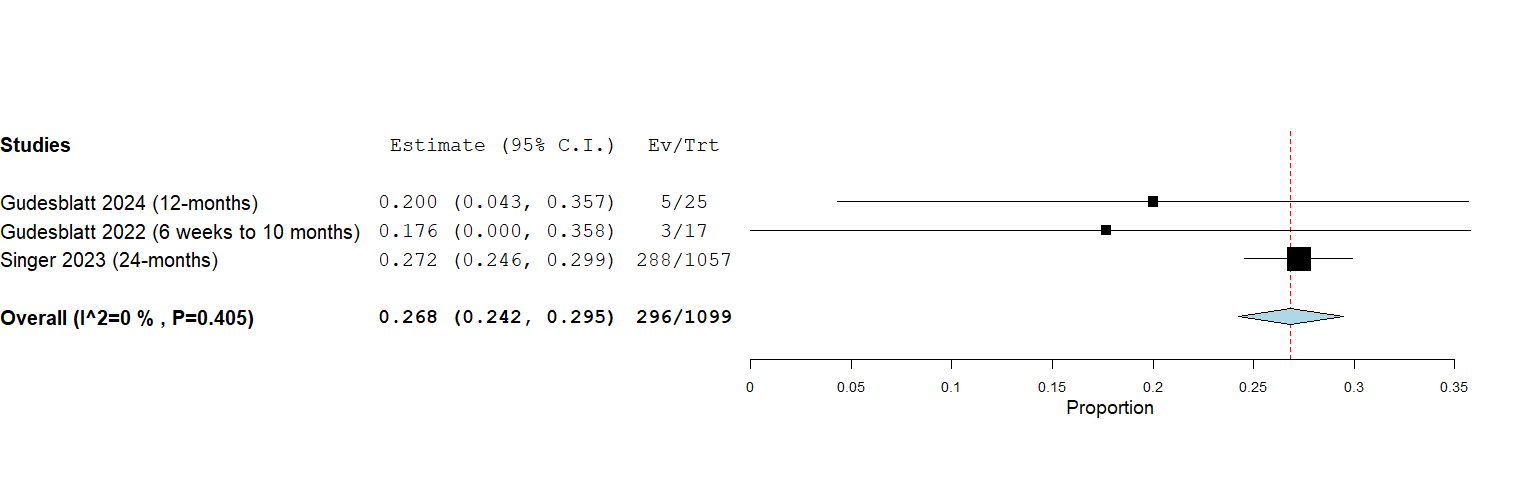


**Supplementary Figure 5.** Leave one out analysis of overall relapse rate in patients taking diroximel fumarate (DRF)


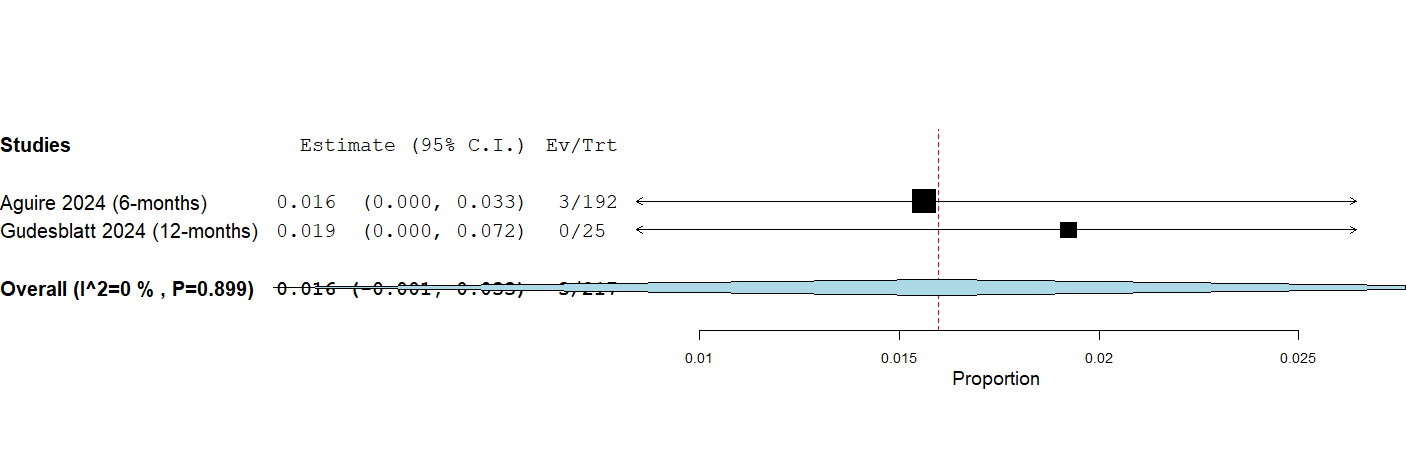

Supplement: Supplementary file 1 — Supplementary file1 (DOCX 90 KB) [file 10072_2025_8140_MOESM1_ESM.docx]
